# LncRNA *MALAT1* Participates in Protection of High-Molecular-Weight Hyaluronan against Smoke-Induced Acute Lung Injury by Upregulation of SOCS-1

**DOI:** 10.3390/molecules27134128

**Published:** 2022-06-27

**Authors:** Shaoguang Li, Bin Li, Ke Lang, Yubei Gong, Xiang Cheng, Shufen Deng, Qiwen Shi, Hang Zhao

**Affiliations:** Collaborative Innovation Center of Yangtze River Delta Region Green Pharmaceuticals, Zhejiang University of Technology, Hangzhou 310014, China; wilsonlsg323@163.com (S.L.); libinzj@foxmail.com (B.L.); langlang6973@163.com (K.L.); gongyubei@foxmail.com (Y.G.); cxtcgtcl@126.com (X.C.); dsfyxj@163.com (S.D.)

**Keywords:** hyaluronan, smoke-induced ALI, metastasis-associated lung adenocarcinoma transcript 1, suppressor of cytokine signaling-1

## Abstract

Smoke-induced acute lung injury (ALI) is a grievous disease with high mortality. Despite advances in medical intervention, no drug has yet been approved by the Food and Drug Administration (FDA) for ALI. In this study, we reported that pretreatment with high-molecular-weight hyaluronan (1600 kDa, HA1600) alleviated pulmonary inflammation and injury in mice exposed to smoke and also upregulated long non-coding RNA (lncRNA) metastasis-associated lung adenocarcinoma transcript 1 (*MALAT1*), as well as suppressor of cytokine signaling-1 (SOCS-1), in the lung tissues. Next, we overexpressed *MALAT1* in the lungs by intratracheal administration of adenovirus cloned with *MALAT1* cDNA and found that the survival of mice after smoke exposure was improved. Moreover, pulmonary overexpression of *MALAT1* ameliorated smoke-induced ALI in mice and elevated the level of SOCS-1 in the lungs. In conclusion, the results pointed out that HA1600 exerted a protective effect against smoke-induced ALI through increasing the *MALAT1* level and the subsequent SOCS-1 expression. Our study provides a potential therapeutic approach to smoke-induced ALI and a novel insight into the mechanism of action of HA1600.

## 1. Introduction

Fire hazard is the leading cause of burn-related injuries, responsible for more than 3,000,000 deaths globally each year [1]. In fact, the smoke released from fire, rather than fire itself, is the most lethal factor [2]. In the summer of 2019, bush fires in southeastern Australia raged for several months, causing millions of people to suffer from lung diseases associated with toxic smoke exposure [3,4,5].

Inhalation of toxic smoke, which is composed of carbon monoxide, cyanide, sulfide, and other hazardous substances, is the primary cause of pulmonary diseases, such as acute lung injury (ALI) and acute respiratory distress syndrome (ARDS) [6,7]. Although advanced treatments have been applied, the mortality of ALI/ARDS is still around 38% [8,9]. Although improved treatments, such as extracorporeal membrane oxygenation (ECMO) and high-frequency percussive ventilation (HEPV), have been developed to alleviate ALI/ARDS, the expense is too high to afford [10,11,12,13]. Moreover, the development of many drugs, including β2 adrenoceptor agonists, keratinocyte growth factor, and statins, terminated during phase II clinical trials because of safety problems [14,15,16,17]. Therefore, there is an urgent need to find a solution for ALI/ARDS.

High-molecular-weight hyaluronan (HMW HA, >1000 kDa) is a straight-chain mucopolysaccharide component of the extracellular matrix (ECM) typically found in healthy tissue. HMW HA has been reported to exert protective effects against inflammation, cell injury, and apoptosis [18,19]. HA with a weight of 1600 kDa (HA1600), but not low-molecular-weight (LMW) HA (35 kDa), blocks neutrophil and monocyte infiltration in lipopolysaccharide (LPS)- and mechanical ventilation-induced ALI [20]. In addition, HMW HA alleviates fine particulate matter (PM_2.5_)-induced ALI by suppressing reactive oxygen species (ROS) in the apoptosis signal-regulating kinase 1 (ASK1)-p38/c-Jun N-terminal kinase (JNK) pathway to reduce pulmonary epithelial apoptosis and inhibiting macrophage M1 polarization to limit the secretion of pro-inflammatory cytokines [21,22]. Based on the previous studies conducted in our group, it is highly possible that HA1600 is also a promising therapeutic strategy for smoke inhalation-induced ALI.

Long non-coding RNAs (lncRNAs) are classified as transcripts over 200 nucleotides in length, and they participate in a variety of cellular processes, including cell differentiation, invasion, migration, and apoptosis [23,24,25]. LncRNA metastasis-associated lung adenocarcinoma transcript 1 (*MALAT1*) was originally identified as a biomarker for lung cancer metastasis [26]. Recent research has revealed that *MALAT1* is highly expressed in almost all tissues and participates in a variety of physiological functions, including neural development, skeletal myogenesis, vascular growth, and inflammation [27,28,29,30,31,32]. However, the role of *MALAT1* in smoke-induced ALI is still unknown.

Suppressor of cytokine signaling-1 (SOCS-1) is an anti-apoptotic and anti-inflammatory protein. Overexpression of SOCS-1 inhibits tumor necrosis factor-α (TNF-α) production and signal transducer and activator of transcription-3 (STAT-3) activation in LPS-induced ALI and represses immune cell accumulation, lung edema, and alveolar dysfunction induced by hyperoxia [33,34]. Consistently, our previous research has demonstrated that, in smoke-induced ALI, SOCS-1 prevents epithelial cell apoptosis by inhibiting the formation of the death-inducing signaling complex (DISC) and ameliorates inflammation through suppressing the assembly of neutrophilic alkaline phosphatase-3 (NALP3) inflammasome in macrophages [35,36].

In the present study, we reported that HA1600 prevented smoke-induced ALI and downregulation of *MALAT1*, and the overexpression of *MALAT1* by adenovirus in the lung exhibited protective effects against smoke-induced ALI and upregulated the protein level of SOCS-1, providing a novel insight into the therapeutic application and mechanism of action of HA1600.

## 2. Results

### 2.1. Intratracheal Nebulization with HA1600 Alleviated Smoke-Induced Acute Lung Injury

**Figure 1:**
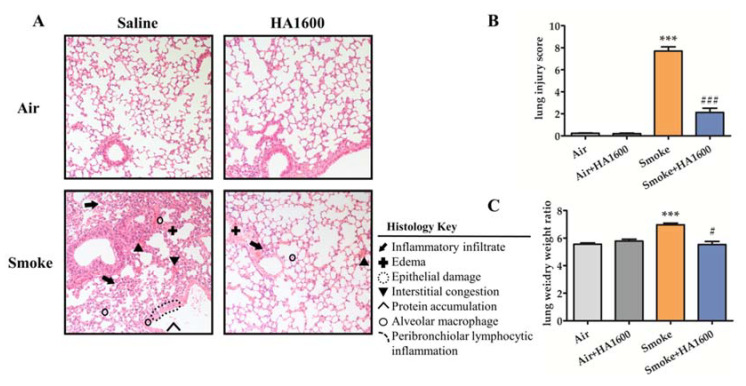
Pretreatment with 1600 kDa hyaluronan (HA1600) protected mice from smoke-induced acute lung injury and inhibited smoke-induced lung edema. Mice were exposed to smoke for 15 min after intratracheal nebulization with HA1600. After 24 h for recovery, mice were sacrificed. (**A**) The lung tissue section stained by hematoxylin and eosin (H&E) (magnification 200×). (**B**) The lung injury scores were calculated for randomly selected area of staining sections. (**C**) The lung wet-to-dry weight ratio was determined. Data are represented as means ± SEM (*n* = 6). *** *p* < 0.001 vs. Air group (the control group); ### *p* < 0.001, # *p* < 0.01 vs. Smoke group.

To evaluate the therapeutic effect of HA1600, C57BL/6J mice intratracheally administrated with HA1600 or normal saline (NS) were put in the smoke chamber and sacrificed 24 h later to collect lung tissues for hematoxylin and eosin (H&E) staining. As shown in Figure 1A, the lung sections of smoke-exposed mice exhibited serious damage compared with those of the control group, characterized by interstitial congestion, edema, inflammatory infiltration, and protein accumulation. By comparison, mice pretreated with HA1600 displayed fewer pathological changes in the lungs after smoke exposure. Next, we quantitated the damage score of histopathology sections according to the criteria described in Table 1. 

The score of the Smoke+HA1600 group was remarkably lower than the Smoke group (Figure 1B). Lung edema was measured as the lung wet-to-dry weight ratio. Consistent with the histological analysis, mice exposed to smoke had more serious edema compared with the control group, and the administration of HA1600 blocked the development of smoke-induced lung edema (Figure 1C). Taken together, HA1600 exerted protective action in smoke-induced ALI.

### 2.2. HA1600 Inhibited Smoke-Induced Pulmonary Inflammation

**Figure 2:**
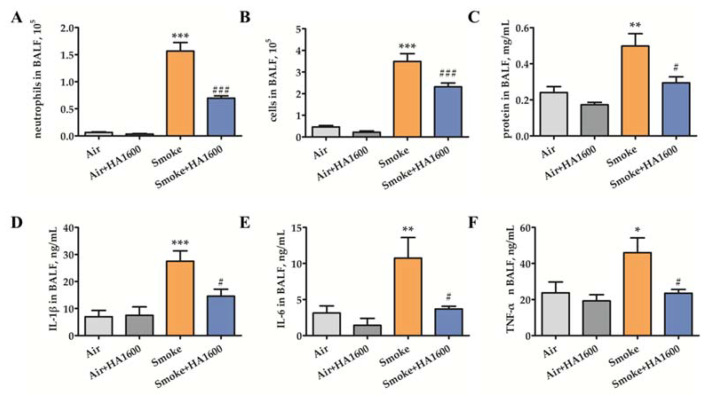
Intratracheal nebulization with HA1600 inhibited pulmonary inflammation after smoke exposure. (**A**) Numbers of neutrophils in bronchoalveolar lavage fluid (BALF) were determined by Wright-Giemsa staining. (**B**) Total cells in BALF were counted. (**C**) Total protein level in BALF was measured by Bicinchoninic Acid (BCA) Protein Assay Kit. The level of inflammatory cytokines in BALF, including (**D**) interleukin (IL)-1β, (**E**) IL-6, and (**F**) tumor necrosis factor (TNF)-α, was detected by enzyme-linked immunosorbent assay (ELISA) kit. Data are represented as means ± SEM (*n* = 6). *** *p* < 0.001, ** *p* < 0.01, and * *p* < 0.05 vs. Air group (the control group); ### *p* < 0.001, # *p* < 0.05 vs. Smoke group.

To assess the effect of HA1600 against smoke-induced pulmonary inflammation, the bronchoalveolar lavage fluid (BALF) was collected for further evaluation. The numbers of neutrophils and total cells in BALF were counted. As illustrated in Figure 2A,B, smoke exposure resulted in a marked increase in the numbers of neutrophils and total cells, which was reversed by HA1600 pretreatment. Alveolar integrity was measured by protein effusion in BALF. The result showed a decrease in protein concentration in the Smoke+HA1600 group compared with the Smoke group, indicating that the administration of HA1600 protected against a smoke-induced pathological deficiency of membrane integrity (Figure 2C). The levels of interleukin (IL)-1β, IL-6, and tumor necrosis factor (TNF)-α in BALF were also detected as indicators of inflammation infiltration. The level of IL-1β in the Smoke group was higher than that in the control group, and HA1600 administration resulted in a lower production of IL-1β (Figure 2D). Similar results were observed in the levels of IL-6 and TNF-α (Figure 2E,F). In sum, our data pointed out that HA1600 protected against smoke-induced diffuse alveolar damage (DAD) and the following lung inflammation response.

### 2.3. Intratracheal Nebulization with HA1600 Increased MALAT1 Expression in Smoke-Induced ALI

**Figure 3:**
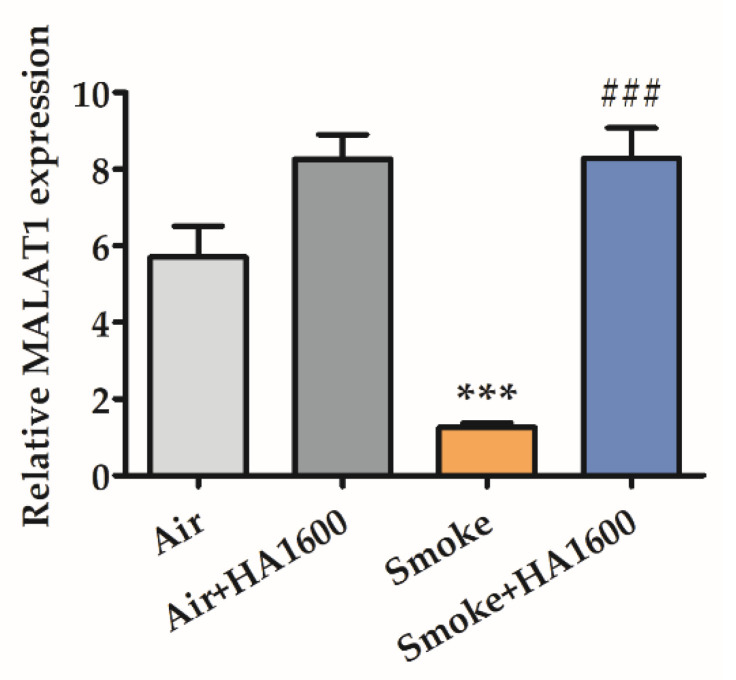
HA1600 increased metastasis-associated lung adenocarcinoma transcript 1 (*MALAT1*) expression in smoke-induced ALI. Mice were allowed to recover after smoke exposure for 24 h, and the level of *MALAT1* in lung tissues was analyzed by quantitative real-time (RT) PCR. Data are represented as means ± SEM (*n* = 6). *** *p* < 0.001 vs. Air group (the control group); ### *p* < 0.001 vs. Smoke group.

It is reported that *MALAT1* sequesters the nuclear factor kappa-B (NF-κB) subunits p65 and p50 in the nucleus and then weakens the following subset of NF-κB driven promoters, such as TNF-α and IL-6 [37]. The above data proved the beneficial therapy of HA1600 through the inhibition of smoke-induced lung inflammation. Thus, we hypothesized that HA1600 might regulate *MALAT1* expression to exert a curative effect. To determine the effect of HA1600 on lncRNA *MALAT1*, different groups of mice were anesthetized and administered with NS and HA1600 separately through intratracheal administration 30 min before exposure with or without smoke. After 24 h of recovery, the mice were sacrificed, and the whole lung was removed for quantitative real-time (RT) polymerase chain reaction (PCR) analysis. As shown in Figure 3, the *MALAT1* expression after smoke exposure was decreased three-fold compared with the control groups, and the reduction was reversed by nebulization with HA1600. Nevertheless, this result was not sufficient to prove the role of *MALAT1* in ALI. Thus, we intended to explore whether *MALAT1* affected smoke inhalation-induced ALI in the following experiments.

### 2.4. Overexpression of MALAT1 in Lung Tissue Prolonged Animal Survival and Alleviated Lung Damage after Smoke Exposure

**Figure 4:**
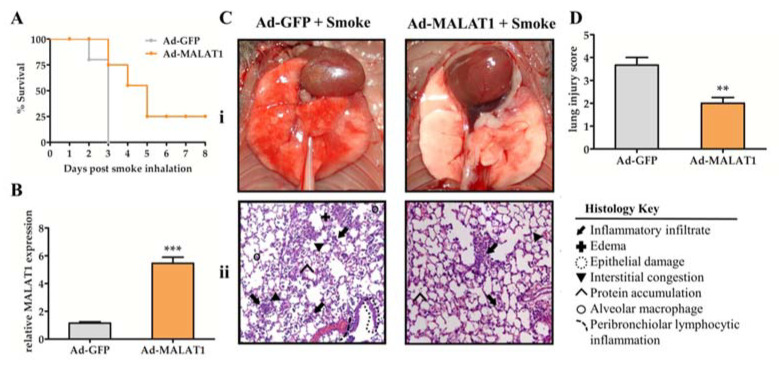
Overexpression of *MALAT1* prolonged survival and alleviated lung damage after smoke inhalation. (**A**) Adenovirus *MALAT1* (Ad-*MALAT1*) or adenovirus green fluorescent protein (Ad-GFP) was delivered into mice’s lungs 3 days before the smoke exposure. After 15 min smoke inhalation, the survival was monitored for up to 8 days (*n* = 20). (**B**) Verification of adenoviral transfection in the lungs tissues was determined by quantitative RT-PCR analysis. (**C**) Lung injury observation (i) of each group was performed, and the lung tissue sections stained by H&E (ii) were observed under morphological alteration (magnification 200×). (**D**) Lung injury scores were calculated from random area of H&E staining sections. Data are represented as means ± SEM (*n* = 6). *** *p* < 0.001, ** *p* < 0.01 vs. Ad-GFP group.

According to Cai et al., knockdown *MALAT1* in human fibroblasts WI-38 cells promotes cell apoptosis [38]. Moreover, data obtained in the study of Li et al. suggest that silencing *MALAT1* aggravates pulmonary microvascular endothelial cell apoptosis, indicating that *MALAT1* exerts a protective role in cell apoptosis and even in innate immune injury [30]. In our study, the full-length mouse *MALAT1* cDNA was cloned into the adenoviral expression vector (Ad-*MALAT1*), and the adenovirus expressing green fluorescent protein (Ad-GFP) was used in the control group. Then, Ad-*MALAT1* or Ad-GFP was delivered to the mice’s lungs by intratracheal nebulization. After animal models had been constructed for 3 days and exposed to smoke for 15 min, the survival was monitored in the following 8 days. It was observed that the mice’s survival rate in the Ad-GFP control group began to decrease on the second day, while all the mice were still alive in the Ad-*MALAT1* group. Later, all Ad-GFP mice died on the third day, and meanwhile, 75% of mice survived in the Ad-*MALAT1* group. Eventually, Ad-*MALAT1* infected mice survived up to 8 days, at which point they were sacrificed, with a survival rate of 25% (Figure 4A). Lung tissue was collected for each group, and the *MALAT1* expression was quantified by quantitative RT-PCR. The results verified the successful overexpression of *MALAT1* in the lung tissues (Figure 4B).

To investigate more detailed histological changes in overexpressed mouse models, the H&E staining sections of lungs were prepared, and the lung injury score was evaluated according to Table 1. 

Mice in the Ad-GFP group showed typical lung injury features, including a dark red, moist surface with exudation and diffuse hyperemia (Figure 4C). On the contrary, the lungs from the Ad-*MALAT1* group remained pink without hemorrhage, edema, or exudation. Moreover, neutrophil infiltration, thickening of the alveolar wall, and epithelial damage were remarkably reduced in the lungs from the Ad-*MALAT1* group compared with the Ad-GFP group. Histological analysis revealed mild symptoms and lower scores in mice transfected with Ad-*MALAT1* and exposed to smoke (Figure 4D). Hence, our results confirmed that increased expression of lncRNA *MALAT1* is beneficial to smoke-induced ALI.

### 2.5. Overexpression of MALAT1 Increases the Level of SOCS-1 after Smoke Exposure

**Figure 5:**
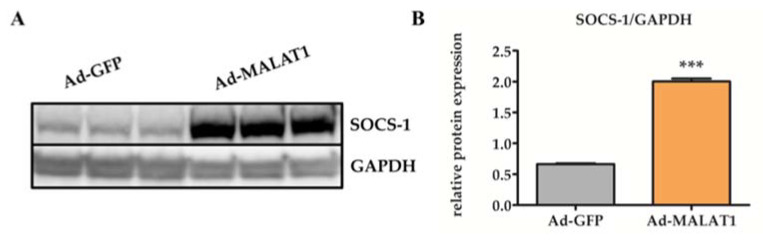
Overexpression of *MALAT1* promoted the expression of suppressor of cytokine signaling-1 (SOCS-1) in the murine lungs after smoke exposure. Mice were transfected by Ad-GFP or Ad-*MALAT1* by intratracheal nebulization and sacrificed one day after smoke inhalation. (**A**) Western blot to analyze SOCS-1 expression in the lungs of mice transfected with Ad-GFP or Ad-*MALAT1* and exposed to smoke. (**B**) Densitometric quantification of the Western blots. Data are represented as means ± SEM (*n* = 6). *** *p* < 0.01 vs. Ad-GFP group.

Previous research proved that SOCS-1 is a negative regulator of proinflammatory cytokine signaling induced by smoke inhalation [36]. We, therefore, hypothesized that *MALAT1* may modulate SOCS-1 expression. To test this possibility, we extracted total lung protein from mice in the Ad-GFP and Ad-*MALAT1* groups the day after smoke exposure. Subsequently, SOCS-1 expression was analyzed by Western blot. As shown in Figure 5A,B, the expression of SOCS-1 in the lung tissues of mice in the Ad-*MALAT1* group was appreciably upregulated. Meanwhile, the SOCS-1 level in the Ad-GFP group was barely increased. It can be inferred that overexpression of *MALAT1* enhances the expression of SOCS-1. Our data indicated that lncRNA *MALAT1* alleviated smoke-induced ALI via upregulating the expression of SOCS-1 in the lungs.

## 3. Discussion

In this study, we identified lncRNA *MALAT1* as a potential target of HA1600 in the treatment of smoke-induced ALI and demonstrated that *MALAT1* is an upstream regulator of SOCS-1. Pretreatment with HA1600 suppressed smoke-induced lung inflammation, edema, and injury and elevated the level of *MALAT1* and SOCS-1 in the murine lungs. Intratracheal nebulization with adenovirus cloned with *MALAT1* cDNA alleviated pulmonary injury and increased the survival rate of mice, as well as SOCS-1 expression, after some exposure. Our results indicated that HA1600 mitigated smoke-induced ALI through the sequential upregulation of *MALAT1* and SOCS-1.

Inhalation injury brings a cascade of pathological damages to burned victims. The rationale behind ALI is the diffuse alveolar damage (DAD) that presents a deficiency of membrane integrity at first. Consequently, the increased permeability of the endothelium and epithelium membrane initiates neutrophil migration, capillary endothelium injury, alveolar epithelium injury, proinflammatory cytokines secretion, and edema [39]. The progression of ALI/ARDS can be aggravated by barrier dysfunction of the capillary endothelium, alveolar epithelium injury, degeneration of pulmonary surfactant, inflammatory infiltration, and bacterial infection [7]. In the present study, we reported that intratracheal administration of HA1600 prevented smoke-induced ALI and inflammation in mice. Consistently, our previous investigation on rats also noticed that pretreatment with HA1600 for 18 h significantly reduced neutrophil infiltration, lung edema, and mucous plugging after smoke exposure [19]. Our preclinical data conclude that HA1600 is effective in the prevention of smoke-induced ALI.

Long non-coding RNA can function as a decoy for transcription factors or as a sponge for microRNA to regulate epigenetic modification. Moreover, an amount of evidence shows that lncRNAs participate in a variety of biological processes, including cell differentiation, apoptosis, proliferation, and even chromatin remodeling [32]. The exudative stage of ALI/ARDS is characterized by injured endothelial cells, which cause cytokine storms and an influx of neutrophils, macrophages, and red blood cells. Migrated neutrophils in the alveoli usually block or repair the gaps of the wall in physiology, whereas aggravated injury of the alveolar wall in pathological conditions causes a positive feedback loop. The role of *MALAT1* in injury and inflammation seems to be controversial. Genetic deletion of *MALAT1* inhibits endothelial cell proliferation, indicating that *MALAT1* may contribute to repairing endothelial cells during the acute phase of ALI and alleviating the following cytokine storms [40]. Sebastian et al. not only reported that *MALAT1* protected mice from atherosclerosis by decreasing cytokine production, but also found that above-median *MALAT1* expression levels in the plaques were associated with fewer major adverse events and better survival rate [41]. In contrast, Liang et al. revealed that knockdown of *MALAT1* in A549 cells downregulated the levels of inflammatory cytokines and inhibited the NF-κB pathway [42]. However, most studies focused on in vitro study instead of animal models. Our study overexpressed *MALAT1* in the whole lung and manifested that the mice administered with Ad-*MALAT1* by intratracheal nebulization were protected from smoke-induced epithelial injury, neutrophil infiltration, and congestion. The result that one-quarter of the mice survived after smoke treatment further confirmed the protective role of *MALAT1* in ALI. Future study may continue with the function of *MALAT1* in different cells, such as macrophages, neutrophils, and epithelium.

Growing evidence shows that lncRNA *MALAT1* participates in the regulation of inflammation. The inflammation, apoptosis, and maturation process of airway epithelial dendritic cells can be regulated by *MALAT1* [29]. Similarly, *MALAT1* targets toll-like receptor-4 (TLR-4) to inhibit inflammatory cytokine secretion, resist epithelial apoptosis, and alleviate ALI via NF-κB/p38 mitogen-activated protein kinase (MAPK) signaling pathway [30]. In line with those studies, our sections and assessments also clearly demonstrate that *MALAT1* regulated inflammation and stages of ALI. The significant reduction in neutrophils observed in our study was consistent with the demonstration that mice transplanted with *MALAT1* deletion bone marrow showed increased numbers of proinflammatory cells and cytokines [41].

SOCS-1, a member of the cytokine signaling pathway inhibitor family, exerts a protective role in cells against apoptosis and inflammation. Our previous study found that overexpression of SOCS-1 increased the survival of mice and relieved smoke-induced ALI through altering the TNF receptor-associated death domain protein (TRADD) and caspase-8 to interfere with the formation of the death-inducing signaling complex [35]. Moreover, smoke inhalation induces NALP3 inflammasome formation, caspase-1 activation, and pulmonary inflammation via adenosine triphosphate (ATP) activating the P2X7 receptor (P2X7R). The combination of P2X7R and ATP causes the enhancement of K^+^ efflux in macrophages, while NALP3 inflammasome assembly and K^+^ efflux can be blocked by SOCS-1, which interferes with the NALP3–apoptosis-associated speck-line protein containing a CARD (ACS) interaction [36]. The caspase-1 activation and excretion of IL-1β could also be inhibited in the same way. Here, we noticed that SOCS-1 was enhanced after smoke exposure via upregulating the level of *MALAT1*, and the control group did not change. The results indicated that SOCS-1, as a downstream target, could be regulated by lncRNA *MALAT1* in smoke inhalation-induced ALI, which would also explain the crucial role of *MALAT1* in HA1600 alleviated lung damage, while whether *MALAT1* suppresses NALP3 inflammasome formation and even the DISC apoptosis pathway deserves to be further investigated. Moreover, Li et al. revealed that *MALAT1* alleviated the ox-low density lipoproteins (ox-LDL)-induced inflammation and apoptosis of Human Coronary Artery Endothelial Cells (HCAECs) via sponging miR-155 to increase the SOCS-1 level [43]. In our previous study, we also proved the relationships of miR-155 and SOCS-1 in smoke-induced ALI mice [44]. Thus, it is worth exploring the underlying mechanism.

The global outbreak of Corona Virus Disease 2019 (COVID-19) causes individual infection and leads to severe pulmonary disease with high mortality. Gratefully, several pioneering studies have confirmed that among the BALF of infected individuals was the *MALAT1*, which showed a significant increase in transcription level [45]. In line with this research, the gene expression of *MALAT1* was upregulated by virus infection in normal human bronchial epithelial (NHBE) cells [46]. We anticipate that *MALAT1* might also act as an agent for the regulation of infiltration to the site of infection. Despite all this emerging research, the role of *MALAT1* in Severe Acute Respiratory Syndrome Coronavirus 2 (SRAS-CoV-2)-induced inflammation storms is still unknown. In this study, we highlight the protective function of lncRNA *MALAT1* and SOCS-1 in vivo. Moreover, researchers have constructed a lethal SARS-CoV-2 mouse model that captured several aspects of symptoms of COVID-19, providing a platform for evaluating antiviral drugs and investigating underlying pathways of ALI/ARDS [47]. Thus, the precise role of *MALAT1* and SOCS-1, whether as a potential therapeutic target of ALI/ARDS, a regulator of inflammation, or a biomarker in COVID-19, needs to be deciphered.

In conclusion, our research is the first to build the connection of lncRNA *MALAT1* and SOCS-1 in HA1600 attenuated smoke-induced ALI. *MALAT1* serves as a downstream target of HA1600, as well as a negative regulator role in smoke-mediated lung neutrophil infiltration, edema, hemorrhage congestion, and interstitial fibroblasts via SOCS-1. Further possible mechanisms, including the activation of caspase-1, -3, or -8, assembly of the NALP3 inflammasome, and the formation of DISC, deserve to be investigated. This study will contribute to the research and development of HA1600 as a candidate drug for curing ALI/ARDS in the foreseeable future.

## 4. Materials and Methods

### 4.1. Animals

C57BL/6J male mice aged 6–8 weeks were supplied by the Zhejiang Academy of Medical Sciences. All animals were maintained under specific pathogen-free (SPF) conditions at a temperature of 24 ± 1 °C under a 12 h light: 12 h dark cycle and fed on a standard diet with water ad libitum. For the care and use of animals, all the animal experiments were handled following the NIH guide for laboratory animals (NIH Publication No. 85-23, revised 1996) and approved by the Laboratory Animal Ethical Committee of the Zhejiang University of Technology (Registered number. 20210707052, 7 July 2021).

### 4.2. Pretreatment with HA1600 and Smoke Inhalation

Mice were randomly divided into four groups, anesthetized, and administrated intratracheally with normal saline (NS) and 0.35% HA1600 (1600 kDa Hyaluronan, Thermo Fisher Scientific, Waltham, MA, USA). Both groups were administrated 30 min before smoke or air exposure (*n* = 6 per set).

Smoke inhalation was administered as previously described [48]. Briefly, the mice were anesthetized, pretreated, and carefully placed in the smoke chamber, which was filled with smoke from 300 mg of smoldering cotton heated to 400 °C, for 15 min. During the period of combustion and smoke exposure, the temperature in the smoke chamber was not allowed to exceed 40 °C to prevent thermal injury to the airways. All mice were returned to the cages and allowed free access to food and water. After recovery for 24 h, all mice were sacrificed via deep anesthesia with exsanguination. The lung was removed en bloc for further analysis.

### 4.3. Bronchoalveolar Lavage Fluid (BALF) and Cell Coounts

Mice were sacrificed 24 h after exposure. A sterile 22-gauge needle was inserted into the trachea, which was exposed through a midline incision. The right lung was clamped at the bronchus to prevent the lavage fluid from entering. BALF was collected from the left lung by instilling 0.5 mL cold phosphate-buffered saline (PBS) 4 times through the incised trachea. A total volume of at least 1.8 mL BALF was received per mouse. Then, after centrifugation of each BALF sample, the supernatant was stored at −80 °C for determination of cytokines, and the cells were resuspended with 0.2 mL PBS for cell counts. Total cell counts were performed using a hemocytometer. Neutrophils were differentiated by using Wright-Giemsa staining (Solarbio, Beijing, China) and calculated on glass slides.

### 4.4. Adenoviral Vectors

For the overexpression experiments, the full-length mouse *MALAT1* cDNA (Genbank accession number AY722410) was cloned into the adenoviral expression vector (Ad-*MALAT1*) (kind gift from Dr. CA Hales, MGH). Cells were treated with Ad-GFP (control) and Ad-*MALAT1*. After reaching confluence, cells were transduced with adenoviral stock containing 10^8^ Plaque-Forming units (PFU) according to the manufacturer’s protocol (Millipore, Billerica, MA, USA). In this approach, the treatment yielded greater than 95% stable transductants within 10–13 days. Subsequently, mice were intraperitoneally anesthetized, and the adenovirus (10^8^ PFU) in 50 μL of PBS was administrated to the trachea with a micro-sprayer (Penn-Century, Wyndmoor, PA, USA). After 3 days, mice were exposed to the smoke chamber and their survival status monitored for 8 days. At 8 days, all mice were executed to determine signs of lung injury on pathology (*n* = 20 per set).

### 4.5. Isolation of RNA and Quantitative Real-Time RT-PCR

For isolation of total RNA, the lung tissue was homogenized in Trizol reagent, and the supernatant was then manipulated according to the manufacturer’s protocol (Invitrogen, Carlsbad, CA, USA). The cDNA was reversely transcribed using SuperScript II RNase H^−^ Reverse Transcriptase (Invitrogen, Carlsbad, CA, USA) from one microgram of total RNA. PCR was performed with the primers of *MALAT1*, 5′-GGCGGAATTGCTGGTAGTTT-3′, 5′-AGCATAGCAGTACACGCCTT-3′; *GAPDH*, 5′-AGGTCGGTGTGAACGGATTTG-3′, 5′-TGTAGACCATGTAGTTGAGGTCA-3′, using SYBR Green PCR Master Mix (Thermo Fisher Scientific, Waltham, MA, USA).

### 4.6. Lung Histology

For observation and evaluation of the signs of ALI on pathology, mice were sacrificed, and the lung was removed. Each lung was infused in 10% buffered formalin, embedded in paraffin, and sectioned at 4 μm thickness. Tissue sections were then dewaxed in ethanol, rehydrated, and stained with hematoxylin and eosin.

### 4.7. Lung Injury Score

Each pathological change was scored according to Table 1. on a scale of 0 to 3, which is based on the following four parts: (1) thickening of alveoli wall; (2) lung hemorrhage; (3) interstitial fibroblasts; (4) inflammatory infiltration. At least five fields were randomly picked for each lung slice.

### 4.8. Enzyme-Linked Immunosorbent Assay (ELISA) and Bicinchoninic Acid (BCA) Assay

The levels of IL-1β, IL-6, and TNF-α were measured using mouse ELISA kit (Thermo Fisher Scientific, Waltham, MA, USA) according to the manufacturer’s protocol. Protein concentration was assessed using BCA protein assay kit (Beyotime, Shanghai, China) according to the manufacturer’s protocol.

### 4.9. Western Blot Analysis

Lung tissue lysates were electrophoresed on sodium dodecyl sulfate-polyacrylamide gel electrophoresis (SDS-PAGE) gels (Bio-rad, Shanghai, China) and transferred to polyvinylidene difluoride (PVDF) membranes (Millipore, Billerica, MA, US). The membranes were washed in 1× phosphate-buffered saline with Tween-20 (PBST) for 20 min and blocked with 5% skim milk at room temperature for 2 h. Then, the blots were incubated with SOCS-1 antibody or glyceraldehyde-3-phosphate dehydrogenase (*GAPDH*) antibody (Santa Cruz Biotechnology, Inc., Santa Cruz, CA, USA) in tris-buffered saline with Tween-20 (TBST) containing 5% skim milk at 4 °C overnight according to the manufacturer’s instruction. After 20 min washing with 1× PBS with Tween-20, the membranes were incubated with the appropriate horseradish peroxidase-conjugated secondary antibodies (Abcam, Cambridge, MA, USA) in TBST containing 5% skim milk at room temperature for 2 h according to the manufacturer’s protocol. Bands were detected by enhanced chemiluminescence (ECL) (GE, Healthcare, Shanghai, China). The integrated density of the bands was analyzed and quantified using the Image J software (Version 1.46r, National Institutes of Health, Bethesda, MD, USA).

### 4.10. Statistical Method

All data were expressed as mean ± SEM. The Student’s *t*-test was used for comparisons between two groups. Values of *p* < 0.05 were considered significant.

## 5. Conclusions

Our results illustrate that *MALAT1* expression is elevated by HA1600, which exerts protective effects on smoke-induced ALI. In addition, overexpressed *MALAT1* mice constructed by adenovirus carried a full-length sequence of *MALAT1* and exhibited less injury under smoke inhalation, which was shown in both the survival rate and histology changes. Mice in the Ad-*MALAT1* group showed slight neutrophil infiltration, hemorrhage, and edema, as well as epithelial damage. Then, the assessment of the lung injury score in the Ad-GFP group showed significantly higher scores than the Ad-*MALAT1* group. Finally, the level of SOCS-1 was upregulated in the overexpressed *MALAT1* mice models.

## Figures and Tables

**Table 1:** Quantitative histopathology score of lung injury.

Tissue	0	1	2	3
Thickening of the alveolar wall	Normal	Thickening in less than 1/3 area	Thickening in 1/3 to 2/3 area	Thickening in more than 2/3 area
Lung hemorrhage	Normal	1–5 alveoli exist at least 5 erythrocytes	5–10 alveoli exist at least 5 erythrocytes	More than 10 alveoli exist at least 5 erythrocytes
Interstitial fibroblasts	No fibroblasts	Fibrin was observed in less than 1/3 area	Fibrin was observed in 1/3 to 2/3 area	Fibrin was observed in more than 2/3 area
Inflammatory infiltration	Infiltrate in less than 5 cells	Infiltrate in 5–10 cells	Infiltrate in 5–10 cells	Infiltrate in more than 20 cells

## Data Availability

The data that support the findings of this study are available from the corresponding author upon reasonable request.

## Sample Availability

Samples of the compounds are available from the authors.
